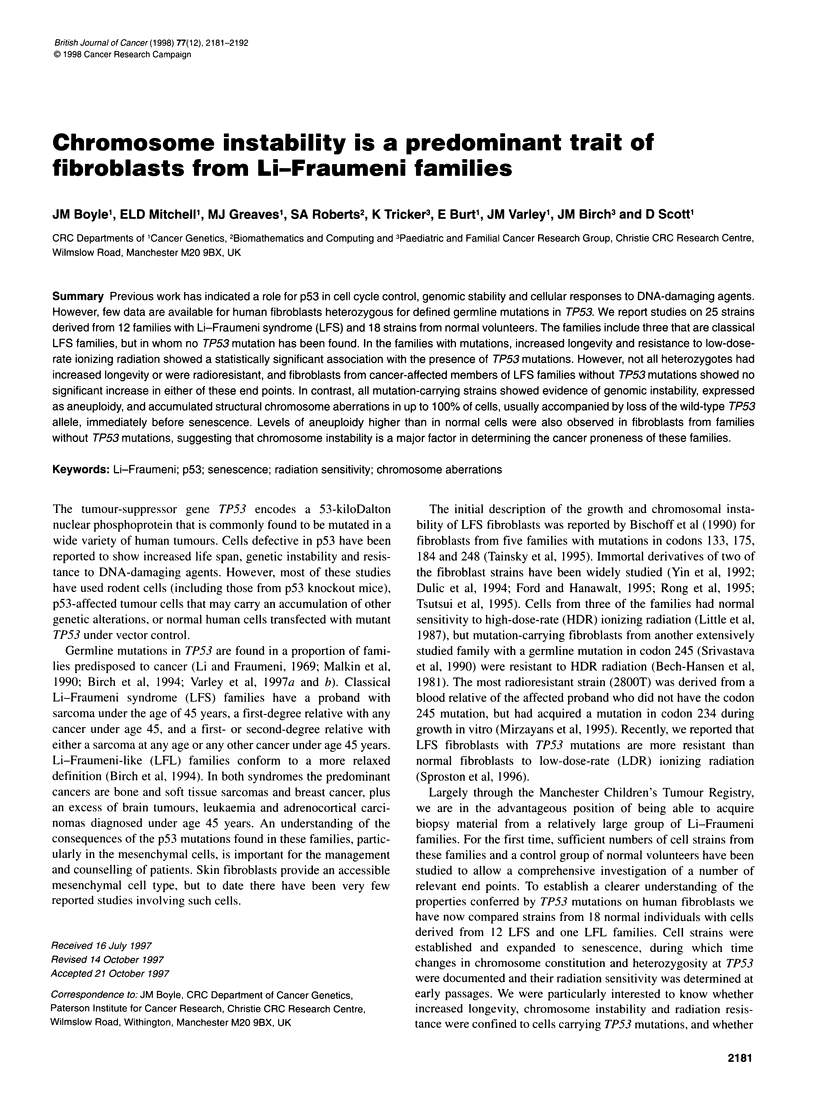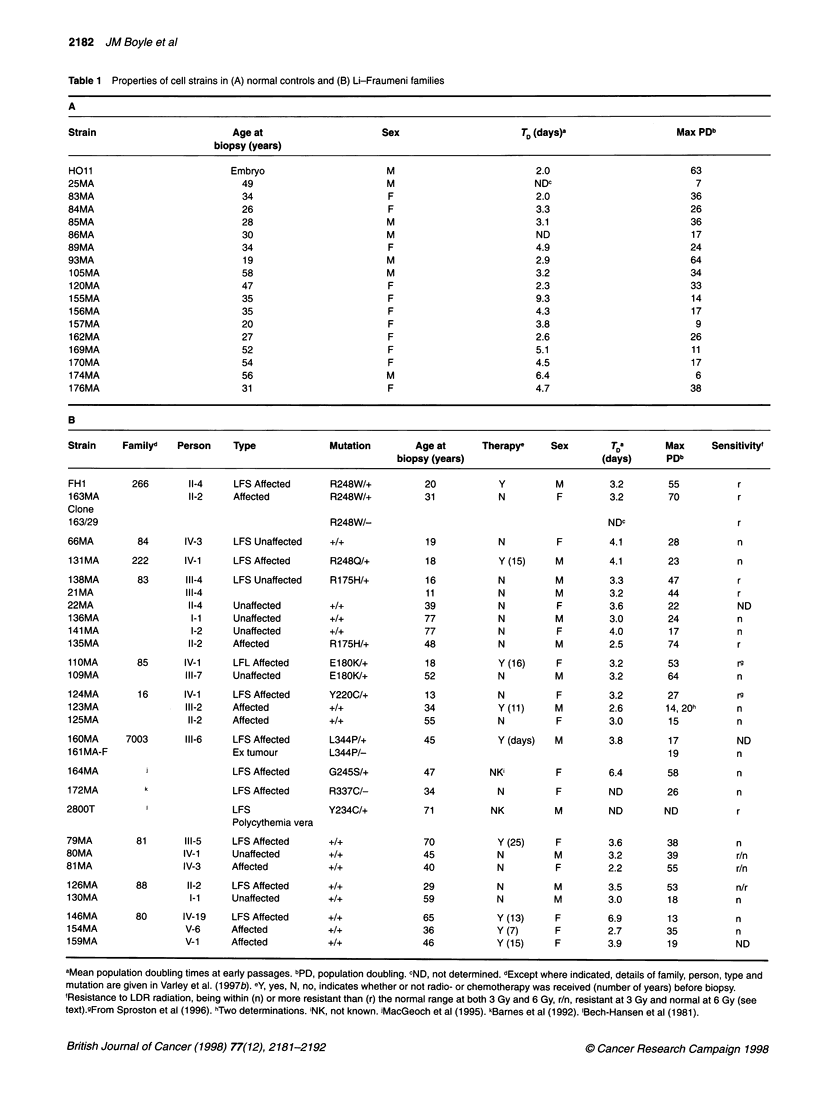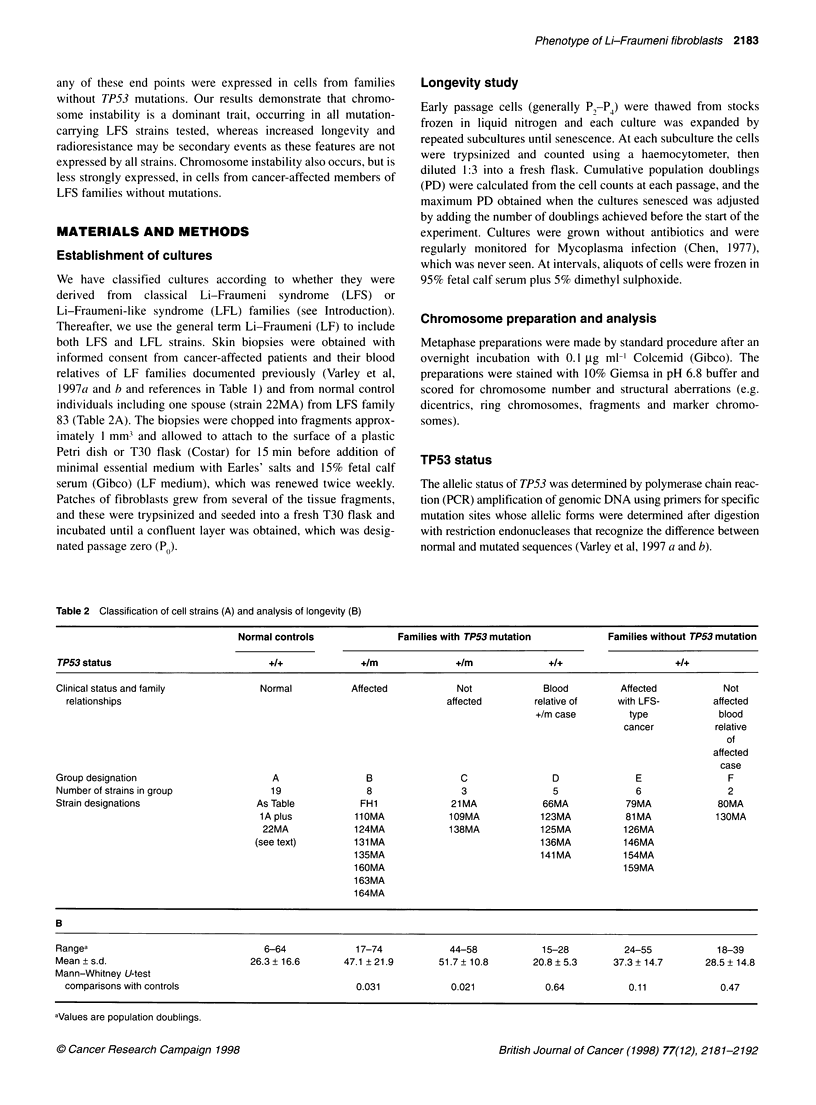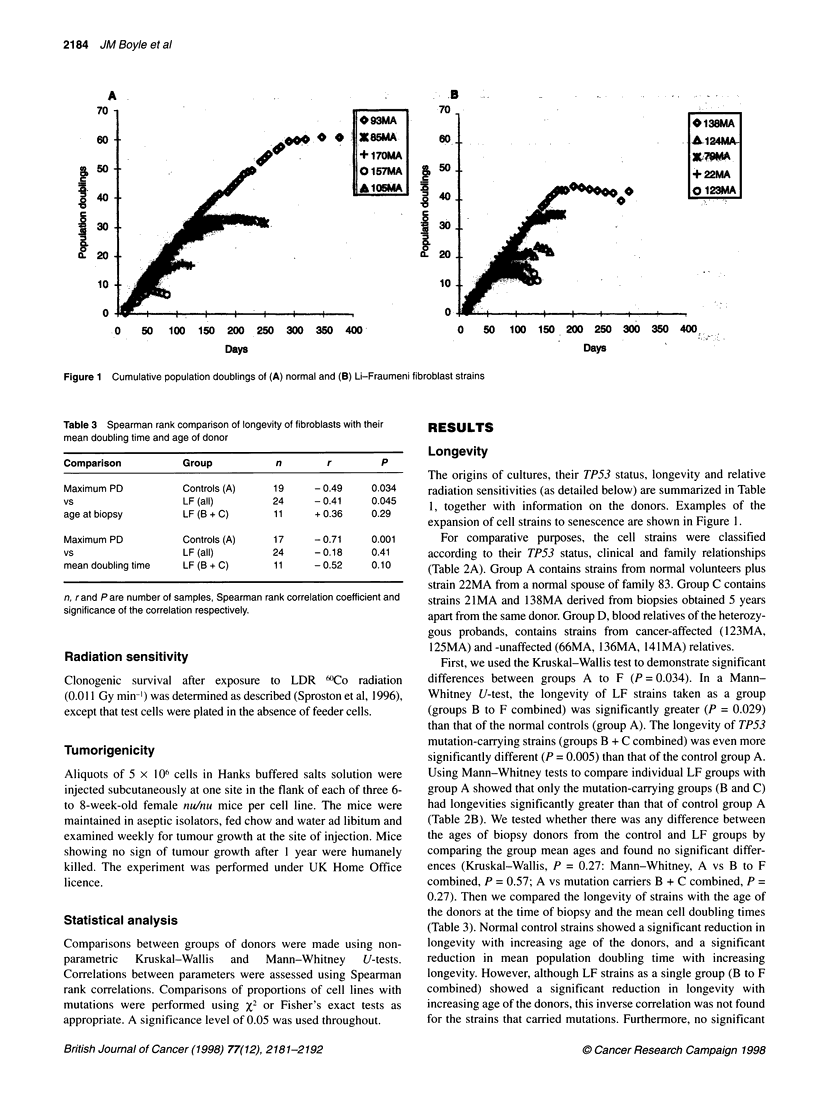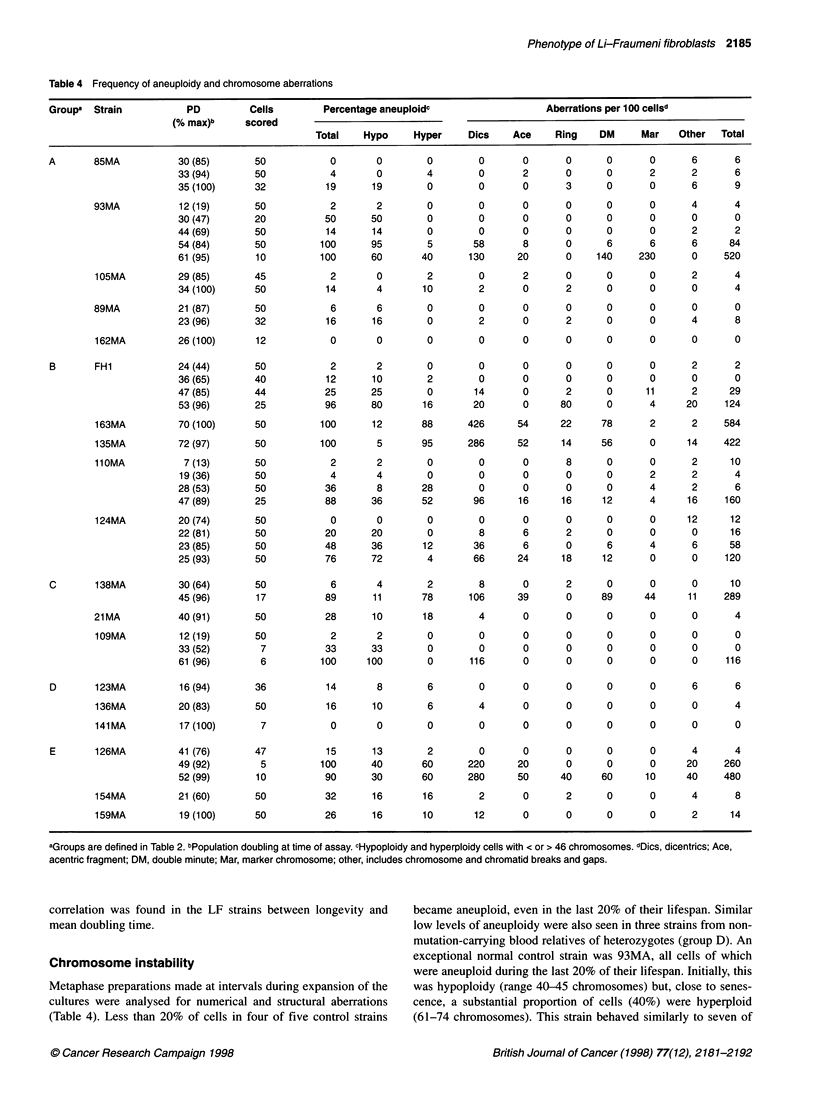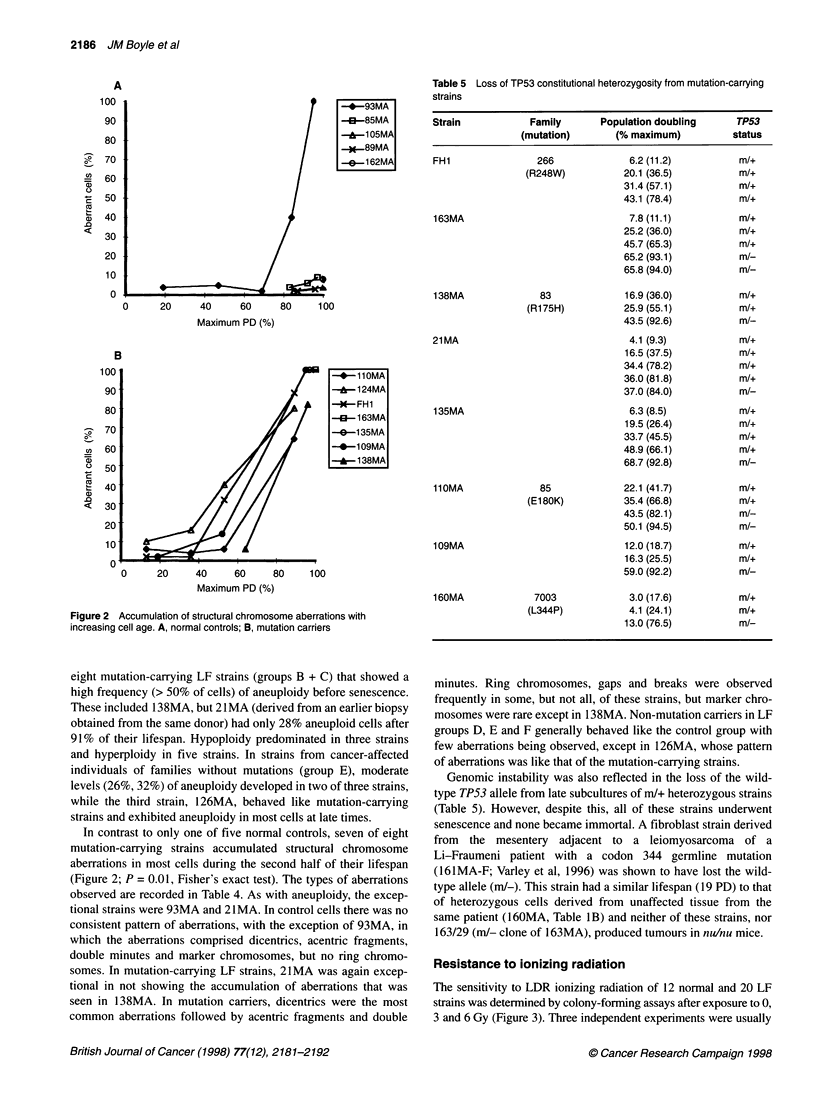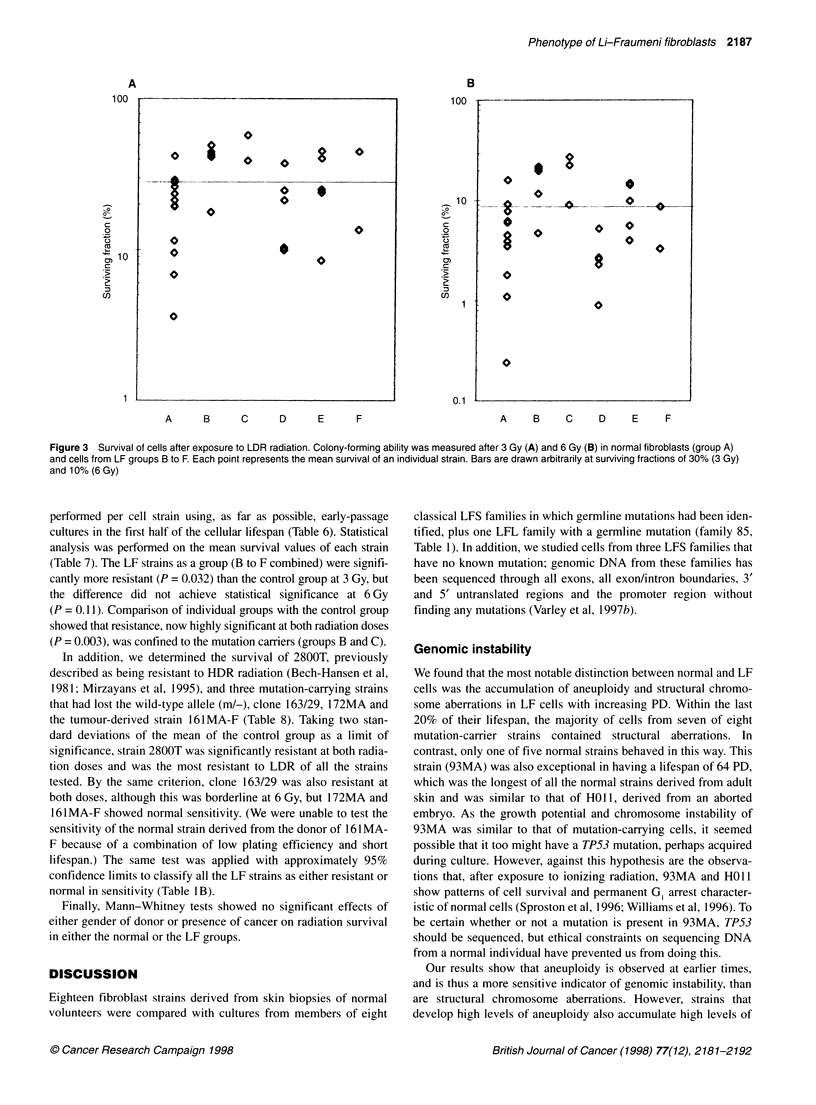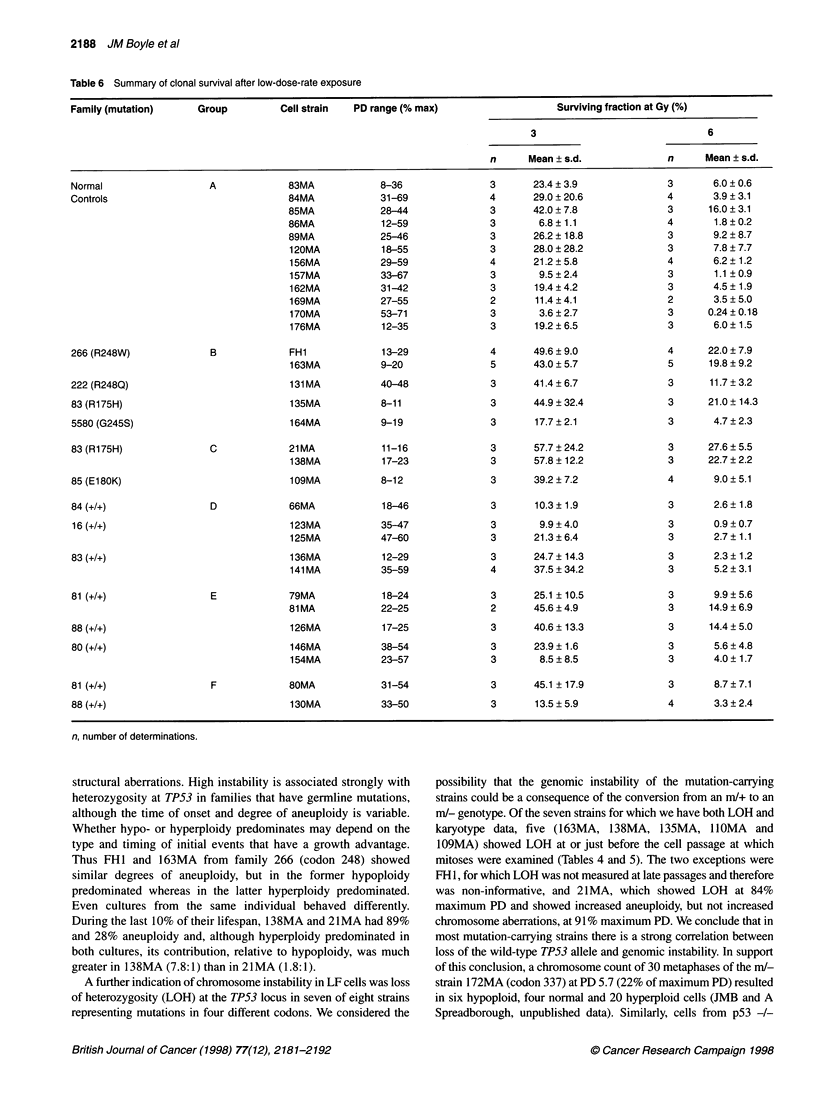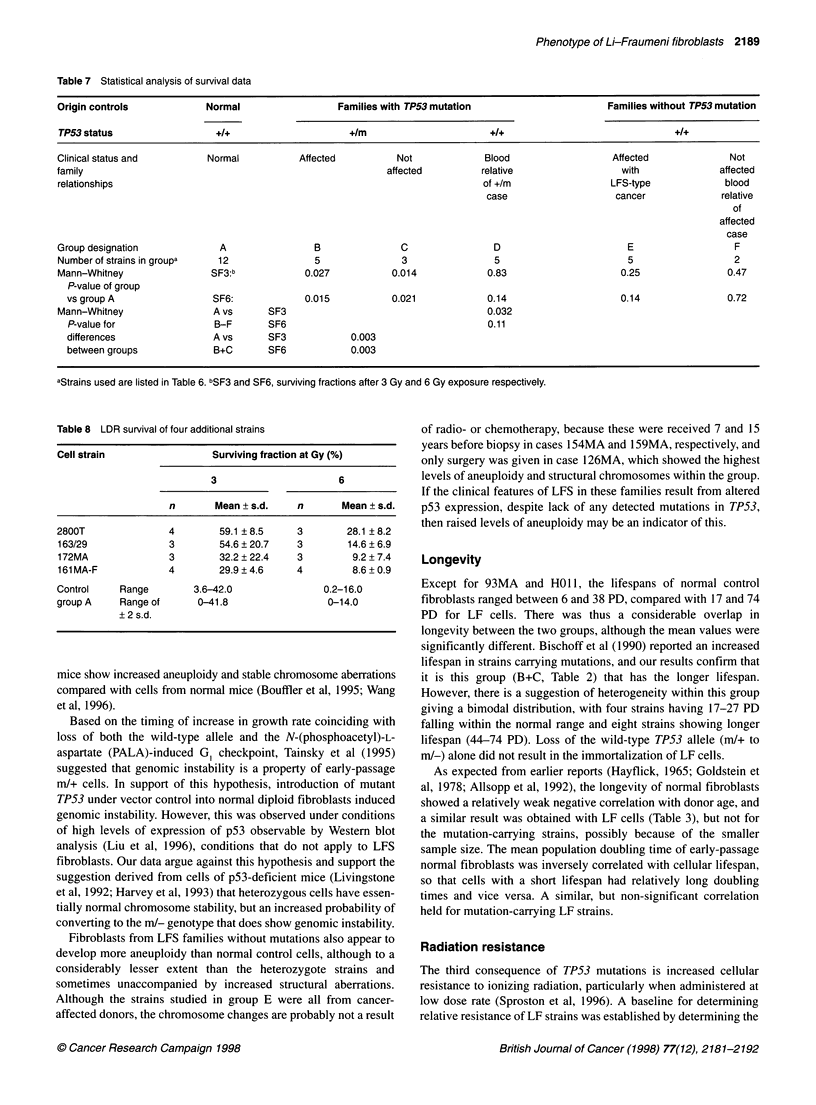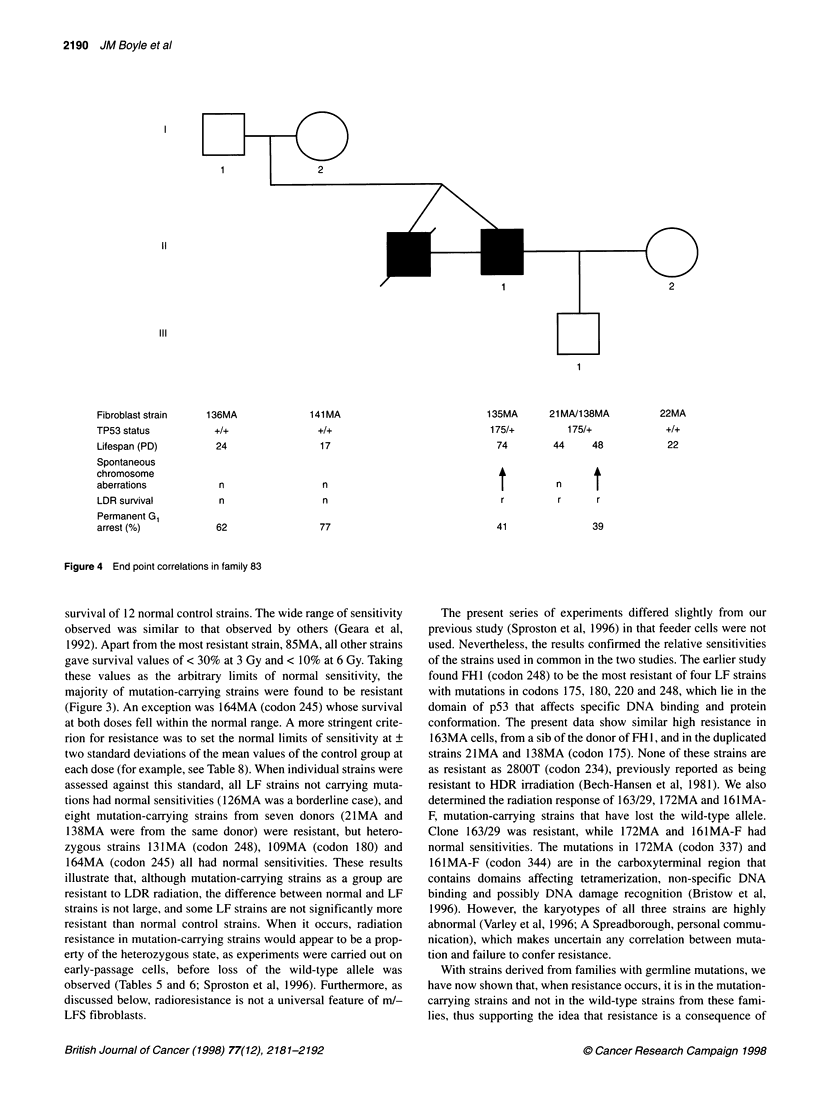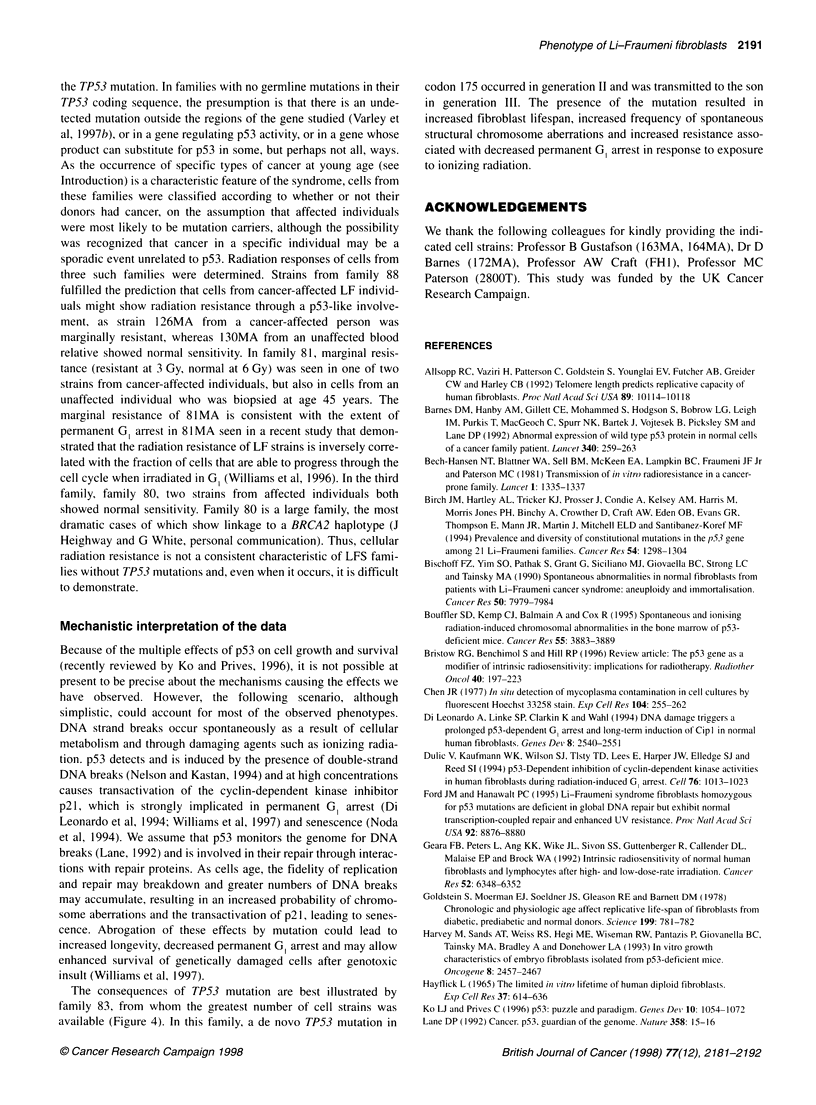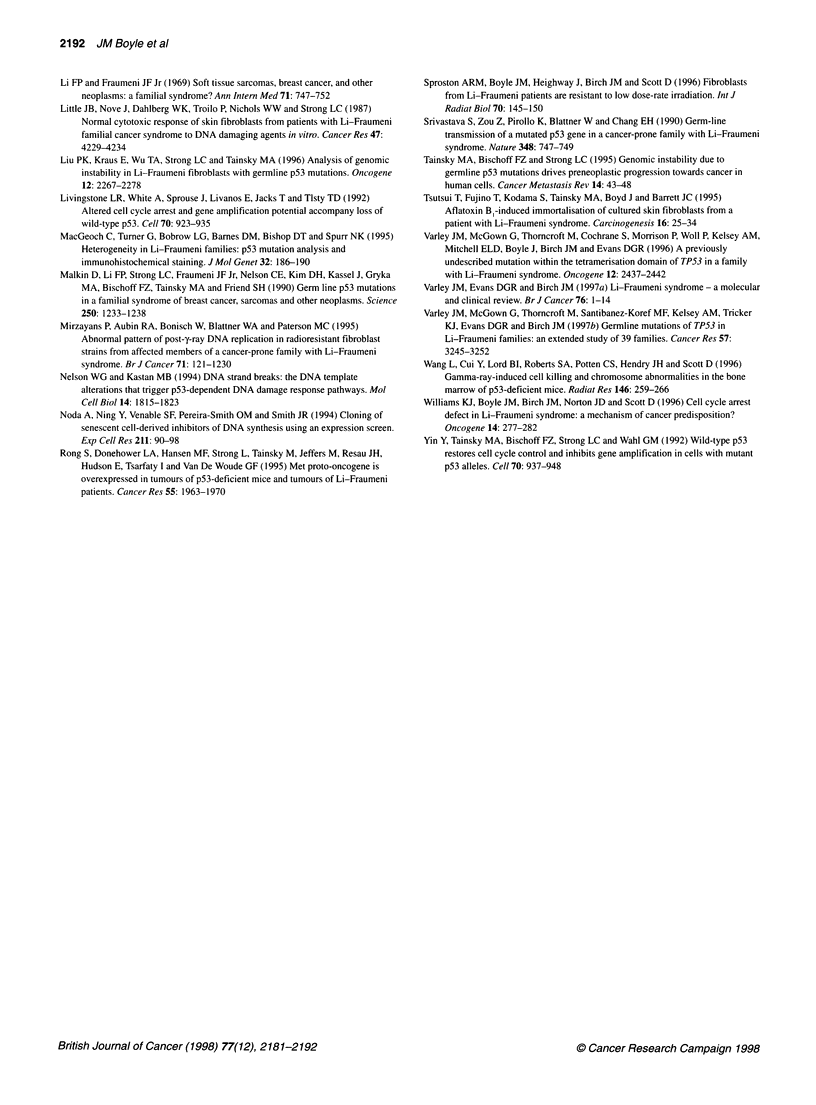# Chromosome instability is a predominant trait of fibroblasts from Li-Fraumeni families.

**DOI:** 10.1038/bjc.1998.364

**Published:** 1998-06

**Authors:** J. M. Boyle, E. L. Mitchell, M. J. Greaves, S. A. Roberts, K. Tricker, E. Burt, J. M. Varley, J. M. Birch, D. Scott

**Affiliations:** CRC Department of Cancer Genetics, Christie CRC Research Centre, Manchester, UK.

## Abstract

Previous work has indicated a role for p53 in cell cycle control, genomic stability and cellular responses to DNA-damaging agents. However, few data are available for human fibroblasts heterozygous for defined germline mutations in TP53. We report studies on 25 strains derived from 12 families with Li-Fraumeni syndrome (LFS) and 18 strains from normal volunteers. The families include three that are classical LFS families, but in whom no TP53 mutation has been found. In the families with mutations, increased longevity and resistance to low-dose-rate ionizing radiation showed a statistically significant association with the presence of TP53 mutations. However, not all heterozygotes had increased longevity or were radioresistant, and fibroblasts from cancer-affected members of LFS families without TP53 mutations showed no significant increase in either of these end points. In contrast, all mutation-carrying strains showed evidence of genomic instability, expressed as aneuploidy, and accumulated structural chromosome aberrations in up to 100% of cells, usually accompanied by loss of the wild-type TP53 allele, immediately before senescence. Levels of aneuploidy higher than in normal cells were also observed in fibroblasts from families without TP53 mutations, suggesting that chromosome instability is a major factor in determining the cancer proneness of these families.


British Journal of Cancer (1998) 77(12), 2181–2192
© 1998 Cancer Research Campaign

### Chromosome instability is a predominant trait of fibroblasts from Li–Fraumeni families

**JM Boyle[1], ELD Mitchell[1], MJ Greaves[1], SA Roberts[2], K Tricker[3], E Burt[1], JM Varley[1], JM Birch[3] and D Scott[1]**

CRC Departments of [1]Cancer Genetics, [2]Biomathematics and Computing and [3]Paediatric and Familial Cancer Research Group, Christie CRC Research Centre, Wilmslow Road, Manchester M20 9BX, UK

**Summary** Previous work has indicated a role for p53 in cell cycle control, genomic stability and cellular responses to DNA-damaging agents. However, few data are available for human fibroblasts heterozygous for defined germline mutations in *TP53*. We report studies on 25 strains derived from 12 families with Li–Fraumeni syndrome (LFS) and 18 strains from normal volunteers. The families include three that are classical LFS families, but in whom no *TP53* mutation has been found. In the families with mutations, increased longevity and resistance to low-dose-rate ionizing radiation showed a statistically significant association with the presence of *TP53* mutations. However, not all heterozygotes had increased longevity or were radioresistant, and fibroblasts from cancer-affected members of LFS families without *TP53* mutations showed no significant increase in either of these end points. In contrast, all mutation-carrying strains showed evidence of genomic instability, expressed as aneuploidy, and accumulated structural chromosome aberrations in up to 100% of cells, usually accompanied by loss of the wild-type *TP53* allele, immediately before senescence. Levels of aneuploidy higher than in normal cells were also observed in fibroblasts from families without *TP53* mutations, suggesting that chromosome instability is a major factor in determining the cancer proneness of these families.

**Keywords:** Li–Fraumeni; p53; senescence; radiation sensitivity; chromosome aberrations

The tumour-suppressor gene *TP53* encodes a 53-kiloDalton nuclear phosphoprotein that is commonly found to be mutated in a wide variety of human tumours. Cells defective in p53 have been reported to show increased life span, genetic instability and resistance to DNA-damaging agents. However, most of these studies have used rodent cells (including those from p53 knockout mice), p53-affected tumour cells that may carry an accumulation of other genetic alterations, or normal human cells transfected with mutant *TP53* under vector control.

Germline mutations in *TP53* are found in a proportion of families predisposed to cancer (Li and Fraumeni, 1969; Malkin et al, 1990; Birch et al, 1994; Varley et al, 1997*a* and *b*). Classical Li–Fraumeni syndrome (LFS) families have a proband with sarcoma under the age of 45 years, a first-degree relative with any cancer under age 45, and a first- or second-degree relative with either a sarcoma at any age or any other cancer under age 45 years. Li–Fraumeni-like (LFL) families conform to a more relaxed definition (Birch et al, 1994). In both syndromes the predominant cancers are bone and soft tissue sarcomas and breast cancer, plus an excess of brain tumours, leukaemia and adrenocortical carcinomas diagnosed under age 45 years. An understanding of the consequences of the p53 mutations found in these families, particularly in the mesenchymal cells, is important for the management and counselling of patients. Skin fibroblasts provide an accessible mesenchymal cell type, but to date there have been very few reported studies involving such cells.

*Received 16 July 1997*
*Revised 14 October 1997*
*Accepted 21 October 1997*

*Correspondence to:* JM Boyle, CRC Department of Cancer Genetics, Paterson Institute for Cancer Research, Christie CRC Research Centre, Wilmslow Road, Withington, Manchester M20 9BX, UK

The initial description of the growth and chromosomal instability of LFS fibroblasts was reported by Bischoff et al (1990) for fibroblasts from five families with mutations in codons 133, 175, 184 and 248 (Tainsky et al, 1995). Immortal derivatives of two of the fibroblast strains have been widely studied (Yin et al, 1992; Dulic et al, 1994; Ford and Hanawalt, 1995; Rong et al, 1995; Tsutsui et al, 1995). Cells from three of the families had normal sensitivity to high-dose-rate (HDR) ionizing radiation (Little et al, 1987), but mutation-carrying fibroblasts from another extensively studied family with a germline mutation in codon 245 (Srivastava et al, 1990) were resistant to HDR radiation (Bech-Hansen et al, 1981). The most radioresistant strain (2800T) was derived from a blood relative of the affected proband who did not have the codon 245 mutation, but had acquired a mutation in codon 234 during growth in vitro (Mirzayans et al, 1995). Recently, we reported that LFS fibroblasts with *TP53* mutations are more resistant than normal fibroblasts to low-dose-rate (LDR) ionizing radiation (Sproston et al, 1996).

Largely through the Manchester Children's Tumour Registry, we are in the advantageous position of being able to acquire biopsy material from a relatively large group of Li–Fraumeni families. For the first time, sufficient numbers of cell strains from these families and a control group of normal volunteers have been studied to allow a comprehensive investigation of a number of relevant end points. To establish a clearer understanding of the properties conferred by *TP53* mutations on human fibroblasts we have now compared strains from 18 normal individuals with cells derived from 12 LFS and one LFL families. Cell strains were established and expanded to senescence, during which time changes in chromosome constitution and heterozygosity at *TP53* were documented and their radiation sensitivity was determined at early passages. We were particularly interested to know whether increased longevity, chromosome instability and radiation resistance were confined to cells carrying *TP53* mutations, and whether

**Table 1** Properties of cell strains in (A) normal controls and (B) Li–Fraumeni families

**A**

| Strain | Age at biopsy (years) | Sex | $T_D$ (days)[a] | Max PD[b] |
|---|---|---|---|---|
| HO11 | Embryo | M | 2.0 | 63 |
| 25MA | 49 | M | ND[c] | 7 |
| 83MA | 34 | F | 2.0 | 36 |
| 84MA | 26 | F | 3.3 | 26 |
| 85MA | 28 | M | 3.1 | 36 |
| 86MA | 30 | M | ND | 17 |
| 89MA | 34 | F | 4.9 | 24 |
| 93MA | 19 | M | 2.9 | 64 |
| 105MA | 58 | M | 3.2 | 34 |
| 120MA | 47 | F | 2.3 | 33 |
| 155MA | 35 | F | 9.3 | 14 |
| 156MA | 35 | F | 4.3 | 17 |
| 157MA | 20 | F | 3.8 | 9 |
| 162MA | 27 | F | 2.6 | 26 |
| 169MA | 52 | F | 5.1 | 11 |
| 170MA | 54 | F | 4.5 | 17 |
| 174MA | 56 | M | 6.4 | 6 |
| 176MA | 31 | F | 4.7 | 38 |

**B**

| Strain | Family[d] | Person | Type | Mutation | Age at biopsy (years) | Therapy[e] | Sex | $T_D$[a] (days) | Max PD[b] | Sensitivity[f] |
|---|---|---|---|---|---|---|---|---|---|---|
| FH1 | 266 | II-4 | LFS Affected | R248W/+ | 20 | Y | M | 3.2 | 55 | r |
| 163MA | | II-2 | Affected | R248W/+ | 31 | N | F | 3.2 | 70 | r |
| Clone 163/29 | | | | R248W/– | | | | ND[c] | | r |
| 66MA | 84 | IV-3 | LFS Unaffected | +/+ | 19 | N | F | 4.1 | 28 | n |
| 131MA | 222 | IV-1 | LFS Affected | R248Q/+ | 18 | Y (15) | M | 4.1 | 23 | n |
| 138MA | 83 | III-4 | LFS Unaffected | R175H/+ | 16 | N | M | 3.3 | 47 | r |
| 21MA | | III-4 | | | 11 | N | M | 3.2 | 44 | r |
| 22MA | | II-4 | Unaffected | +/+ | 39 | N | F | 3.6 | 22 | ND |
| 136MA | | I-1 | Unaffected | +/+ | 77 | N | M | 3.0 | 24 | n |
| 141MA | | I-2 | Unaffected | +/+ | 77 | N | F | 4.0 | 17 | n |
| 135MA | | II-2 | Affected | R175H/+ | 48 | N | M | 2.5 | 74 | r |
| 110MA | 85 | IV-1 | LFL Affected | E180K/+ | 18 | Y (16) | F | 3.2 | 53 | r[g] |
| 109MA | | III-7 | Unaffected | E180K/+ | 52 | N | M | 3.2 | 64 | n |
| 124MA | 16 | IV-1 | LFS Affected | Y220C/+ | 13 | N | F | 3.2 | 27 | r[g] |
| 123MA | | III-2 | Affected | +/+ | 34 | Y (11) | M | 2.6 | 14, 20[h] | n |
| 125MA | | II-2 | Affected | +/+ | 55 | N | F | 3.0 | 15 | n |
| 160MA | 7003 | III-6 | LFS Affected | L344P/+ | 45 | Y (days) | M | 3.8 | 17 | ND |
| 161MA-F | | | Ex tumour | L344P/– | | | | | 19 | n |
| 164MA | [j] | | LFS Affected | G245S/+ | 47 | NK[i] | F | 6.4 | 58 | n |
| 172MA | [k] | | LFS Affected | R337C/– | 34 | N | F | ND | 26 | n |
| 2800T | [l] | | LFS Polycythemia vera | Y234C/+ | 71 | NK | M | ND | ND | r |
| 79MA | 81 | III-5 | LFS Affected | +/+ | 70 | Y (25) | F | 3.6 | 38 | n |
| 80MA | | IV-1 | Unaffected | +/+ | 45 | N | M | 3.2 | 39 | r/n |
| 81MA | | IV-3 | Affected | +/+ | 40 | N | F | 2.2 | 55 | r/n |
| 126MA | 88 | II-2 | LFS Affected | +/+ | 29 | N | M | 3.5 | 53 | n/r |
| 130MA | | I-1 | Unaffected | +/+ | 59 | N | M | 3.0 | 18 | n |
| 146MA | 80 | IV-19 | LFS Affected | +/+ | 65 | Y (13) | F | 6.9 | 13 | n |
| 154MA | | V-6 | Affected | +/+ | 36 | Y (7) | F | 2.7 | 35 | n |
| 159MA | | V-1 | Affected | +/+ | 46 | Y (15) | F | 3.9 | 19 | ND |

[a]Mean population doubling times at early passages. [b]PD, population doubling. [c]ND, not determined. [d]Except where indicated, details of family, person, type and mutation are given in Varley et al. (1997*b*). [e]Y, yes, N, no, indicates whether or not radio- or chemotherapy was received (number of years) before biopsy. [f]Resistance to LDR radiation, being within (n) or more resistant than (r) the normal range at both 3 Gy and 6 Gy, r/n, resistant at 3 Gy and normal at 6 Gy (see text).[g]From Sproston et al (1996). [h]Two determinations. [i]NK, not known. [j]MacGeoch et al (1995). [k]Barnes et al (1992). [l]Bech-Hansen et al (1981).

 *© Cancer Research Campaign 1998*

any of these end points were expressed in cells from families without *TP53* mutations. Our results demonstrate that chromosome instability is a dominant trait, occurring in all mutation-carrying LFS strains tested, whereas increased longevity and radioresistance may be secondary events as these features are not expressed by all strains. Chromosome instability also occurs, but is less strongly expressed, in cells from cancer-affected members of LFS families without mutations.

### MATERIALS AND METHODS

### Establishment of cultures

We have classified cultures according to whether they were derived from classical Li–Fraumeni syndrome (LFS) or Li–Fraumeni-like syndrome (LFL) families (see Introduction). Thereafter, we use the general term Li–Fraumeni (LF) to include both LFS and LFL strains. Skin biopsies were obtained with informed consent from cancer-affected patients and their blood relatives of LF families documented previously (Varley et al, 1997*a* and *b* and references in Table 1) and from normal control individuals including one spouse (strain 22MA) from LFS family 83 (Table 2A). The biopsies were chopped into fragments approximately 1 $mm^3$ and allowed to attach to the surface of a plastic Petri dish or T30 flask (Costar) for 15 min before addition of minimal essential medium with Earles' salts and 15% fetal calf serum (Gibco) (LF medium), which was renewed twice weekly. Patches of fibroblasts grew from several of the tissue fragments, and these were trypsinized and seeded into a fresh T30 flask and incubated until a confluent layer was obtained, which was designated passage zero ($P_0$).

### Longevity study

Early passage cells (generally $P_2$–$P_4$) were thawed from stocks frozen in liquid nitrogen and each culture was expanded by repeated subcultures until senescence. At each subculture the cells were trypsinized and counted using a haemocytometer, then diluted 1:3 into a fresh flask. Cumulative population doublings (PD) were calculated from the cell counts at each passage, and the maximum PD obtained when the cultures senesced was adjusted by adding the number of doublings achieved before the start of the experiment. Cultures were grown without antibiotics and were regularly monitored for Mycoplasma infection (Chen, 1977), which was never seen. At intervals, aliquots of cells were frozen in 95% fetal calf serum plus 5% dimethyl sulphoxide.

### Chromosome preparation and analysis

Metaphase preparations were made by standard procedure after an overnight incubation with 0.1 $\mu g\ ml^{-1}$ Colcemid (Gibco). The preparations were stained with 10% Giemsa in pH 6.8 buffer and scored for chromosome number and structural aberrations (e.g. dicentrics, ring chromosomes, fragments and marker chromosomes).

### TP53 status

The allelic status of *TP53* was determined by polymerase chain reaction (PCR) amplification of genomic DNA using primers for specific mutation sites whose allelic forms were determined after digestion with restriction endonucleases that recognize the difference between normal and mutated sequences (Varley et al, 1997 *a* and *b*).

**Table 2** Classification of cell strains (A) and analysis of longevity (B)

| | Normal controls | Families with *TP53* mutation | | | Families without *TP53* mutation | |
|---|---|---|---|---|---|---|
| ***TP53* status** | **+/+** | **+/m** | **+/m** | **+/+** | **+/+** | |
| Clinical status and family relationships | Normal | Affected | Not affected | Blood relative of +/m case | Affected with LFS-type cancer | Not affected blood relative of affected case |
| Group designation | A | B | C | D | E | F |
| Number of strains in group | 19 | 8 | 3 | 5 | 6 | 2 |
| Strain designations | As Table 1A plus 22MA (see text) | FH1<br>110MA<br>124MA<br>131MA<br>135MA<br>160MA<br>163MA<br>164MA | 21MA<br>109MA<br>138MA | 66MA<br>123MA<br>125MA<br>136MA<br>141MA | 79MA<br>81MA<br>126MA<br>146MA<br>154MA<br>159MA | 80MA<br>130MA |
| **B** | | | | | | |
| Range[a] | 6–64 | 17–74 | 44–58 | 15–28 | 24–55 | 18–39 |
| Mean ± s.d. | 26.3 ± 16.6 | 47.1 ± 21.9 | 51.7 ± 10.8 | 20.8 ± 5.3 | 37.3 ± 14.7 | 28.5 ± 14.8 |
| Mann–Whitney *U*-test comparisons with controls | | 0.031 | 0.021 | 0.64 | 0.11 | 0.47 |

[a]Values are population doublings.

© Cancer Research Campaign 1998

**Figure 1** Cumulative population doublings of (**A**) normal and (**B**) Li–Fraumeni fibroblast strains

**Table 3** Spearman rank comparison of longevity of fibroblasts with their mean doubling time and age of donor

| Comparison | Group | *n* | *r* | *P* |
|---|---|---|---|---|
| Maximum PD | Controls (A) | 19 | − 0.49 | 0.034 |
| vs | LF (all) | 24 | − 0.41 | 0.045 |
| age at biopsy | LF (B + C) | 11 | + 0.36 | 0.29 |
| Maximum PD | Controls (A) | 17 | − 0.71 | 0.001 |
| vs | LF (all) | 24 | − 0.18 | 0.41 |
| mean doubling time | LF (B + C) | 11 | − 0.52 | 0.10 |

*n, r* and *P* are number of samples, Spearman rank correlation coefficient and significance of the correlation respectively.

### Radiation sensitivity

Clonogenic survival after exposure to LDR $^{60}Co$ radiation (0.011 Gy min$^{-1}$) was determined as described (Sproston et al, 1996), except that test cells were plated in the absence of feeder cells.

### Tumorigenicity

Aliquots of $5 \times 10^6$ cells in Hanks buffered salts solution were injected subcutaneously at one site in the flank of each of three 6- to 8-week-old female *nu/nu* mice per cell line. The mice were maintained in aseptic isolators, fed chow and water ad libitum and examined weekly for tumour growth at the site of injection. Mice showing no sign of tumour growth after 1 year were humanely killed. The experiment was performed under UK Home Office licence.

### Statistical analysis

Comparisons between groups of donors were made using non-parametric Kruskal–Wallis and Mann–Whitney *U*-tests. Correlations between parameters were assessed using Spearman rank correlations. Comparisons of proportions of cell lines with mutations were performed using $\chi^2$ or Fisher's exact tests as appropriate. A significance level of 0.05 was used throughout.

### RESULTS

### Longevity

The origins of cultures, their *TP53* status, longevity and relative radiation sensitivities (as detailed below) are summarized in Table 1, together with information on the donors. Examples of the expansion of cell strains to senescence are shown in Figure 1.

For comparative purposes, the cell strains were classified according to their *TP53* status, clinical and family relationships (Table 2A). Group A contains strains from normal volunteers plus strain 22MA from a normal spouse of family 83. Group C contains strains 21MA and 138MA derived from biopsies obtained 5 years apart from the same donor. Group D, blood relatives of the heterozygous probands, contains strains from cancer-affected (123MA, 125MA) and -unaffected (66MA, 136MA, 141MA) relatives.

First, we used the Kruskal-Wallis test to demonstrate significant differences between groups A to F ($P = 0.034$). In a Mann–Whitney *U*-test, the longevity of LF strains taken as a group (groups B to F combined) was significantly greater ($P = 0.029$) than that of the normal controls (group A). The longevity of *TP53* mutation-carrying strains (groups B + C combined) was even more significantly different ($P = 0.005$) than that of the control group A. Using Mann–Whitney tests to compare individual LF groups with group A showed that only the mutation-carrying groups (B and C) had longevities significantly greater than that of control group A (Table 2B). We tested whether there was any difference between the ages of biopsy donors from the control and LF groups by comparing the group mean ages and found no significant differences (Kruskal-Wallis, $P = 0.27$: Mann–Whitney, A vs B to F combined, $P = 0.57$; A vs mutation carriers B + C combined, $P = 0.27$). Then we compared the longevity of strains with the age of the donors at the time of biopsy and the mean cell doubling times (Table 3). Normal control strains showed a significant reduction in longevity with increasing age of the donors, and a significant reduction in mean population doubling time with increasing longevity. However, although LF strains as a single group (B to F combined) showed a significant reduction in longevity with increasing age of the donors, this inverse correlation was not found for the strains that carried mutations. Furthermore, no significant

© Cancer Research Campaign 1998

**Table 4** Frequency of aneuploidy and chromosome aberrations

| Group[a] | Strain | PD (% max)[b] | Cells scored | Percentage aneuploid[c] | | | Aberrations per 100 cells[d] | | | | | | |
|---|---|---|---|---|---|---|---|---|---|---|---|---|---|
| | | | | Total | Hypo | Hyper | Dics | Ace | Ring | DM | Mar | Other | Total |
| A | 85MA | 30 (85) | 50 | 0 | 0 | 0 | 0 | 0 | 0 | 0 | 0 | 6 | 6 |
| | | 33 (94) | 50 | 4 | 0 | 4 | 0 | 2 | 0 | 0 | 2 | 2 | 6 |
| | | 35 (100) | 32 | 19 | 19 | 0 | 0 | 0 | 3 | 0 | 0 | 6 | 9 |
| | 93MA | 12 (19) | 50 | 2 | 2 | 0 | 0 | 0 | 0 | 0 | 0 | 4 | 4 |
| | | 30 (47) | 20 | 50 | 50 | 0 | 0 | 0 | 0 | 0 | 0 | 0 | 0 |
| | | 44 (69) | 50 | 14 | 14 | 0 | 0 | 0 | 0 | 0 | 0 | 2 | 2 |
| | | 54 (84) | 50 | 100 | 95 | 5 | 58 | 8 | 0 | 6 | 6 | 6 | 84 |
| | | 61 (95) | 10 | 100 | 60 | 40 | 130 | 20 | 0 | 140 | 230 | 0 | 520 |
| | 105MA | 29 (85) | 45 | 2 | 0 | 2 | 0 | 2 | 0 | 0 | 0 | 2 | 4 |
| | | 34 (100) | 50 | 14 | 4 | 10 | 2 | 0 | 2 | 0 | 0 | 0 | 4 |
| | 89MA | 21 (87) | 50 | 6 | 6 | 0 | 0 | 0 | 0 | 0 | 0 | 0 | 0 |
| | | 23 (96) | 32 | 16 | 16 | 0 | 2 | 0 | 2 | 0 | 0 | 4 | 8 |
| | 162MA | 26 (100) | 12 | 0 | 0 | 0 | 0 | 0 | 0 | 0 | 0 | 0 | 0 |
| B | FH1 | 24 (44) | 50 | 2 | 2 | 0 | 0 | 0 | 0 | 0 | 0 | 2 | 2 |
| | | 36 (65) | 40 | 12 | 10 | 2 | 0 | 0 | 0 | 0 | 0 | 0 | 0 |
| | | 47 (85) | 44 | 25 | 25 | 0 | 14 | 0 | 2 | 0 | 11 | 2 | 29 |
| | | 53 (96) | 25 | 96 | 80 | 16 | 20 | 0 | 80 | 0 | 4 | 20 | 124 |
| | 163MA | 70 (100) | 50 | 100 | 12 | 88 | 426 | 54 | 22 | 78 | 2 | 2 | 584 |
| | 135MA | 72 (97) | 50 | 100 | 5 | 95 | 286 | 52 | 14 | 56 | 0 | 14 | 422 |
| | 110MA | 7 (13) | 50 | 2 | 2 | 0 | 0 | 0 | 8 | 0 | 0 | 2 | 10 |
| | | 19 (36) | 50 | 4 | 4 | 0 | 0 | 0 | 0 | 0 | 2 | 2 | 4 |
| | | 28 (53) | 50 | 36 | 8 | 28 | 0 | 0 | 0 | 0 | 4 | 2 | 6 |
| | | 47 (89) | 25 | 88 | 36 | 52 | 96 | 16 | 16 | 12 | 4 | 16 | 160 |
| | 124MA | 20 (74) | 50 | 0 | 0 | 0 | 0 | 0 | 0 | 0 | 0 | 12 | 12 |
| | | 22 (81) | 50 | 20 | 20 | 0 | 8 | 6 | 2 | 0 | 0 | 0 | 16 |
| | | 23 (85) | 50 | 48 | 36 | 12 | 36 | 6 | 0 | 6 | 4 | 6 | 58 |
| | | 25 (93) | 50 | 76 | 72 | 4 | 66 | 24 | 18 | 12 | 0 | 0 | 120 |
| C | 138MA | 30 (64) | 50 | 6 | 4 | 2 | 8 | 0 | 2 | 0 | 0 | 0 | 10 |
| | | 45 (96) | 17 | 89 | 11 | 78 | 106 | 39 | 0 | 89 | 44 | 11 | 289 |
| | 21MA | 40 (91) | 50 | 28 | 10 | 18 | 4 | 0 | 0 | 0 | 0 | 0 | 4 |
| | 109MA | 12 (19) | 50 | 2 | 2 | 0 | 0 | 0 | 0 | 0 | 0 | 0 | 0 |
| | | 33 (52) | 7 | 33 | 33 | 0 | 0 | 0 | 0 | 0 | 0 | 0 | 0 |
| | | 61 (96) | 6 | 100 | 100 | 0 | 116 | 0 | 0 | 0 | 0 | 0 | 116 |
| D | 123MA | 16 (94) | 36 | 14 | 8 | 6 | 0 | 0 | 0 | 0 | 0 | 6 | 6 |
| | 136MA | 20 (83) | 50 | 16 | 10 | 6 | 4 | 0 | 0 | 0 | 0 | 0 | 4 |
| | 141MA | 17 (100) | 7 | 0 | 0 | 0 | 0 | 0 | 0 | 0 | 0 | 0 | 0 |
| E | 126MA | 41 (76) | 47 | 15 | 13 | 2 | 0 | 0 | 0 | 0 | 0 | 4 | 4 |
| | | 49 (92) | 5 | 100 | 40 | 60 | 220 | 20 | 0 | 0 | 0 | 20 | 260 |
| | | 52 (99) | 10 | 90 | 30 | 60 | 280 | 50 | 40 | 60 | 10 | 40 | 480 |
| | 154MA | 21 (60) | 50 | 32 | 16 | 16 | 2 | 0 | 2 | 0 | 0 | 4 | 8 |
| | 159MA | 19 (100) | 50 | 26 | 16 | 10 | 12 | 0 | 0 | 0 | 0 | 2 | 14 |

[a]Groups are defined in Table 2. [b]Population doubling at time of assay. [c]Hypoploidy and hyperploidy cells with < or > 46 chromosomes. [d]Dics, dicentrics; Ace, acentric fragment; DM, double minute; Mar, marker chromosome; other, includes chromosome and chromatid breaks and gaps.

correlation was found in the LF strains between longevity and mean doubling time.

### Chromosome instability

Metaphase preparations made at intervals during expansion of the cultures were analysed for numerical and structural aberrations (Table 4). Less than 20% of cells in four of five control strains became aneuploid, even in the last 20% of their lifespan. Similar low levels of aneuploidy were also seen in three strains from non-mutation-carrying blood relatives of heterozygotes (group D). An exceptional normal control strain was 93MA, all cells of which were aneuploid during the last 20% of their lifespan. Initially, this was hypoploidy (range 40–45 chromosomes) but, close to senescence, a substantial proportion of cells (40%) were hyperploid (61–74 chromosomes). This strain behaved similarly to seven of

© Cancer Research Campaign 1998 

**Figure 2** Accumulation of structural chromosome aberrations with increasing cell age. **A**, normal controls; **B**, mutation carriers

**Table 5** Loss of TP53 constitutional heterozygosity from mutation-carrying strains

| Strain | Family (mutation) | Population doubling (% maximum) | *TP53* status |
|---|---|---|---|
| FH1 | 266 | 6.2 (11.2) | m/+ |
| | (R248W) | 20.1 (36.5) | m/+ |
| | | 31.4 (57.1) | m/+ |
| | | 43.1 (78.4) | m/+ |
| 163MA | | 7.8 (11.1) | m/+ |
| | | 25.2 (36.0) | m/+ |
| | | 45.7 (65.3) | m/+ |
| | | 65.2 (93.1) | m/– |
| | | 65.8 (94.0) | m/– |
| 138MA | 83 | 16.9 (36.0) | m/+ |
| | (R175H) | 25.9 (55.1) | m/+ |
| | | 43.5 (92.6) | m/– |
| 21MA | | 4.1 (9.3) | m/+ |
| | | 16.5 (37.5) | m/+ |
| | | 34.4 (78.2) | m/+ |
| | | 36.0 (81.8) | m/+ |
| | | 37.0 (84.0) | m/– |
| 135MA | | 6.3 (8.5) | m/+ |
| | | 19.5 (26.4) | m/+ |
| | | 33.7 (45.5) | m/+ |
| | | 48.9 (66.1) | m/+ |
| | | 68.7 (92.8) | m/– |
| 110MA | 85 | 22.1 (41.7) | m/+ |
| | (E180K) | 35.4 (66.8) | m/+ |
| | | 43.5 (82.1) | m/– |
| | | 50.1 (94.5) | m/– |
| 109MA | | 12.0 (18.7) | m/+ |
| | | 16.3 (25.5) | m/+ |
| | | 59.0 (92.2) | m/– |
| 160MA | 7003 | 3.0 (17.6) | m/+ |
| | (L344P) | 4.1 (24.1) | m/+ |
| | | 13.0 (76.5) | m/– |

eight mutation-carrying LF strains (groups B + C) that showed a high frequency (> 50% of cells) of aneuploidy before senescence. These included 138MA, but 21MA (derived from an earlier biopsy obtained from the same donor) had only 28% aneuploid cells after 91% of their lifespan. Hypoploidy predominated in three strains and hyperploidy in five strains. In strains from cancer-affected individuals of families without mutations (group E), moderate levels (26%, 32%) of aneuploidy developed in two of three strains, while the third strain, 126MA, behaved like mutation-carrying strains and exhibited aneuploidy in most cells at late times.

In contrast to only one of five normal controls, seven of eight mutation-carrying strains accumulated structural chromosome aberrations in most cells during the second half of their lifespan (Figure 2; $P = 0.01$, Fisher's exact test). The types of aberrations observed are recorded in Table 4. As with aneuploidy, the exceptional strains were 93MA and 21MA. In control cells there was no consistent pattern of aberrations, with the exception of 93MA, in which the aberrations comprised dicentrics, acentric fragments, double minutes and marker chromosomes, but no ring chromosomes. In mutation-carrying LF strains, 21MA was again exceptional in not showing the accumulation of aberrations that was seen in 138MA. In mutation carriers, dicentrics were the most common aberrations followed by acentric fragments and double minutes. Ring chromosomes, gaps and breaks were observed frequently in some, but not all, of these strains, but marker chromosomes were rare except in 138MA. Non-mutation carriers in LF groups D, E and F generally behaved like the control group with few aberrations being observed, except in 126MA, whose pattern of aberrations was like that of the mutation-carrying strains.

Genomic instability was also reflected in the loss of the wild-type *TP53* allele from late subcultures of m/+ heterozygous strains (Table 5). However, despite this, all of these strains underwent senescence and none became immortal. A fibroblast strain derived from the mesentery adjacent to a leiomyosarcoma of a Li–Fraumeni patient with a codon 344 germline mutation (161MA-F; Varley et al, 1996) was shown to have lost the wild-type allele (m/–). This strain had a similar lifespan (19 PD) to that of heterozygous cells derived from unaffected tissue from the same patient (160MA, Table 1B) and neither of these strains, nor 163/29 (m/– clone of 163MA), produced tumours in *nu/nu* mice.

### Resistance to ionizing radiation

The sensitivity to LDR ionizing radiation of 12 normal and 20 LF strains was determined by colony-forming assays after exposure to 0, 3 and 6 Gy (Figure 3). Three independent experiments were usually

© Cancer Research Campaign 1998

**Figure 3** Survival of cells after exposure to LDR radiation. Colony-forming ability was measured after 3 Gy (**A**) and 6 Gy (**B**) in normal fibroblasts (group A) and cells from LF groups B to F. Each point represents the mean survival of an individual strain. Bars are drawn arbitrarily at surviving fractions of 30% (3 Gy) and 10% (6 Gy)

performed per cell strain using, as far as possible, early-passage cultures in the first half of the cellular lifespan (Table 6). Statistical analysis was performed on the mean survival values of each strain (Table 7). The LF strains as a group (B to F combined) were significantly more resistant ($P = 0.032$) than the control group at 3 Gy, but the difference did not achieve statistical significance at 6 Gy ($P = 0.11$). Comparison of individual groups with the control group showed that resistance, now highly significant at both radiation doses ($P = 0.003$), was confined to the mutation carriers (groups B and C).

In addition, we determined the survival of 2800T, previously described as being resistant to HDR radiation (Bech-Hansen et al, 1981; Mirzayans et al, 1995), and three mutation-carrying strains that had lost the wild-type allele (m/–), clone 163/29, 172MA and the tumour-derived strain 161MA-F (Table 8). Taking two standard deviations of the mean of the control group as a limit of significance, strain 2800T was significantly resistant at both radiation doses and was the most resistant to LDR of all the strains tested. By the same criterion, clone 163/29 was also resistant at both doses, although this was borderline at 6 Gy, but 172MA and 161MA-F showed normal sensitivity. (We were unable to test the sensitivity of the normal strain derived from the donor of 161MA-F because of a combination of low plating efficiency and short lifespan.) The same test was applied with approximately 95% confidence limits to classify all the LF strains as either resistant or normal in sensitivity (Table 1B).

Finally, Mann–Whitney tests showed no significant effects of either gender of donor or presence of cancer on radiation survival in either the normal or the LF groups.

### DISCUSSION

Eighteen fibroblast strains derived from skin biopsies of normal volunteers were compared with cultures from members of eight classical LFS families in which germline mutations had been identified, plus one LFL family with a germline mutation (family 85, Table 1). In addition, we studied cells from three LFS families that have no known mutation; genomic DNA from these families has been sequenced through all exons, all exon/intron boundaries, 3′ and 5′ untranslated regions and the promoter region without finding any mutations (Varley et al, 1997*b*).

### Genomic instability

We found that the most notable distinction between normal and LF cells was the accumulation of aneuploidy and structural chromosome aberrations in LF cells with increasing PD. Within the last 20% of their lifespan, the majority of cells from seven of eight mutation-carrier strains contained structural aberrations. In contrast, only one of five normal strains behaved in this way. This strain (93MA) was also exceptional in having a lifespan of 64 PD, which was the longest of all the normal strains derived from adult skin and was similar to that of H011, derived from an aborted embryo. As the growth potential and chromosome instability of 93MA was similar to that of mutation-carrying cells, it seemed possible that it too might have a *TP53* mutation, perhaps acquired during culture. However, against this hypothesis are the observations that, after exposure to ionizing radiation, 93MA and H011 show patterns of cell survival and permanent $G_1$ arrest characteristic of normal cells (Sproston et al, 1996; Williams et al, 1996). To be certain whether or not a mutation is present in 93MA, *TP53* should be sequenced, but ethical constraints on sequencing DNA from a normal individual have prevented us from doing this.

Our results show that aneuploidy is observed at earlier times, and is thus a more sensitive indicator of genomic instability, than are structural chromosome aberrations. However, strains that develop high levels of aneuploidy also accumulate high levels of

© Cancer Research Campaign 1998

**Table 6** Summary of clonal survival after low-dose-rate exposure

| Family (mutation) | Group | Cell strain | PD range (% max) | Surviving fraction at Gy (%) | | | |
|---|---|---|---|---|---|---|---|
| | | | | 3 | | 6 | |
| | | | | *n* | Mean ± s.d. | *n* | Mean ± s.d. |
| Normal Controls | A | 83MA | 8–36 | 3 | 23.4 ± 3.9 | 3 | 6.0 ± 0.6 |
| | | 84MA | 31–69 | 4 | 29.0 ± 20.6 | 4 | 3.9 ± 3.1 |
| | | 85MA | 28–44 | 3 | 42.0 ± 7.8 | 3 | 16.0 ± 3.1 |
| | | 86MA | 12–59 | 3 | 6.8 ± 1.1 | 4 | 1.8 ± 0.2 |
| | | 89MA | 25–46 | 3 | 26.2 ± 18.8 | 3 | 9.2 ± 8.7 |
| | | 120MA | 18–55 | 3 | 28.0 ± 28.2 | 3 | 7.8 ± 7.7 |
| | | 156MA | 29–59 | 4 | 21.2 ± 5.8 | 4 | 6.2 ± 1.2 |
| | | 157MA | 33–67 | 3 | 9.5 ± 2.4 | 3 | 1.1 ± 0.9 |
| | | 162MA | 31–42 | 3 | 19.4 ± 4.2 | 3 | 4.5 ± 1.9 |
| | | 169MA | 27–55 | 2 | 11.4 ± 4.1 | 2 | 3.5 ± 5.0 |
| | | 170MA | 53–71 | 3 | 3.6 ± 2.7 | 3 | 0.24 ± 0.18 |
| | | 176MA | 12–35 | 3 | 19.2 ± 6.5 | 3 | 6.0 ± 1.5 |
| 266 (R248W) | B | FH1 | 13–29 | 4 | 49.6 ± 9.0 | 4 | 22.0 ± 7.9 |
| | | 163MA | 9–20 | 5 | 43.0 ± 5.7 | 5 | 19.8 ± 9.2 |
| 222 (R248Q) | | 131MA | 40–48 | 3 | 41.4 ± 6.7 | 3 | 11.7 ± 3.2 |
| 83 (R175H) | | 135MA | 8–11 | 3 | 44.9 ± 32.4 | 3 | 21.0 ± 14.3 |
| 5580 (G245S) | | 164MA | 9–19 | 3 | 17.7 ± 2.1 | 3 | 4.7 ± 2.3 |
| 83 (R175H) | C | 21MA | 11–16 | 3 | 57.7 ± 24.2 | 3 | 27.6 ± 5.5 |
| | | 138MA | 17–23 | 3 | 57.8 ± 12.2 | 3 | 22.7 ± 2.2 |
| 85 (E180K) | | 109MA | 8–12 | 3 | 39.2 ± 7.2 | 4 | 9.0 ± 5.1 |
| 84 (+/+) | D | 66MA | 18–46 | 3 | 10.3 ± 1.9 | 3 | 2.6 ± 1.8 |
| 16 (+/+) | | 123MA | 35–47 | 3 | 9.9 ± 4.0 | 3 | 0.9 ± 0.7 |
| | | 125MA | 47–60 | 3 | 21.3 ± 6.4 | 3 | 2.7 ± 1.1 |
| 83 (+/+) | | 136MA | 12–29 | 3 | 24.7 ± 14.3 | 3 | 2.3 ± 1.2 |
| | | 141MA | 35–59 | 4 | 37.5 ± 34.2 | 3 | 5.2 ± 3.1 |
| 81 (+/+) | E | 79MA | 18–24 | 3 | 25.1 ± 10.5 | 3 | 9.9 ± 5.6 |
| | | 81MA | 22–25 | 2 | 45.6 ± 4.9 | 3 | 14.9 ± 6.9 |
| 88 (+/+) | | 126MA | 17–25 | 3 | 40.6 ± 13.3 | 3 | 14.4 ± 5.0 |
| 80 (+/+) | | 146MA | 38–54 | 3 | 23.9 ± 1.6 | 3 | 5.6 ± 4.8 |
| | | 154MA | 23–57 | 3 | 8.5 ± 8.5 | 3 | 4.0 ± 1.7 |
| 81 (+/+) | F | 80MA | 31–54 | 3 | 45.1 ± 17.9 | 3 | 8.7 ± 7.1 |
| 88 (+/+) | | 130MA | 33–50 | 3 | 13.5 ± 5.9 | 4 | 3.3 ± 2.4 |

*n*, number of determinations.

structural aberrations. High instability is associated strongly with heterozygosity at *TP53* in families that have germline mutations, although the time of onset and degree of aneuploidy is variable. Whether hypo- or hyperploidy predominates may depend on the type and timing of initial events that have a growth advantage. Thus FH1 and 163MA from family 266 (codon 248) showed similar degrees of aneuploidy, but in the former hypoploidy predominated whereas in the latter hyperploidy predominated. Even cultures from the same individual behaved differently. During the last 10% of their lifespan, 138MA and 21MA had 89% and 28% aneuploidy and, although hyperploidy predominated in both cultures, its contribution, relative to hypoploidy, was much greater in 138MA (7.8:1) than in 21MA (1.8:1).

A further indication of chromosome instability in LF cells was loss of heterozygosity (LOH) at the *TP53* locus in seven of eight strains representing mutations in four different codons. We considered the possibility that the genomic instability of the mutation-carrying strains could be a consequence of the conversion from an m/+ to an m/– genotype. Of the seven strains for which we have both LOH and karyotype data, five (163MA, 138MA, 135MA, 110MA and 109MA) showed LOH at or just before the cell passage at which mitoses were examined (Tables 4 and 5). The two exceptions were FH1, for which LOH was not measured at late passages and therefore was non-informative, and 21MA, which showed LOH at 84% maximum PD and showed increased aneuploidy, but not increased chromosome aberrations, at 91% maximum PD. We conclude that in most mutation-carrying strains there is a strong correlation between loss of the wild-type *TP53* allele and genomic instability. In support of this conclusion, a chromosome count of 30 metaphases of the m/– strain 172MA (codon 337) at PD 5.7 (22% of maximum PD) resulted in six hypoploid, four normal and 20 hyperploid cells (JMB and A Spreadborough, unpublished data). Similarly, cells from p53 –/–

© Cancer Research Campaign 1998

**Table 7** Statistical analysis of survival data

| **Origin controls** | **Normal** | | **Families with *TP53* mutation** | | | **Families without *TP53* mutation** | |
|---|---|---|---|---|---|---|---|
| ***TP53* status** | **+/+** | | **+/m** | | **+/+** | **+/+** | |
| Clinical status and family relationships | Normal | | Affected | Not affected | Blood relative of +/m case | Affected with LFS-type cancer | Not affected blood relative of affected case |
| Group designation | A | | B | C | D | E | F |
| Number of strains in group[a] | 12 | | 5 | 3 | 5 | 5 | 2 |
| Mann–Whitney *P*-value of group vs group A | SF3:[b] | | 0.027 | 0.014 | 0.83 | 0.25 | 0.47 |
| | SF6: | | 0.015 | 0.021 | 0.14 | 0.14 | 0.72 |
| Mann–Whitney *P*-value for differences between groups | A vs B–F | SF3 | | | 0.032 | | |
| | | SF6 | | | 0.11 | | |
| | A vs B+C | SF3 | 0.003 | | | | |
| | | SF6 | 0.003 | | | | |

[a]Strains used are listed in Table 6. [b]SF3 and SF6, surviving fractions after 3 Gy and 6 Gy exposure respectively.

**Table 8** LDR survival of four additional strains

| **Cell strain** | | **Surviving fraction at Gy (%)** | | | |
|---|---|---|---|---|---|
| | | **3** | | **6** | |
| | | ***n*** | **Mean ± s.d.** | ***n*** | **Mean ± s.d.** |
| 2800T | | 4 | 59.1 ± 8.5 | 3 | 28.1 ± 8.2 |
| 163/29 | | 3 | 54.6 ± 20.7 | 3 | 14.6 ± 6.9 |
| 172MA | | 3 | 32.2 ± 22.4 | 3 | 9.2 ± 7.4 |
| 161MA-F | | 4 | 29.9 ± 4.6 | 4 | 8.6 ± 0.9 |
| Control group A | Range | | 3.6–42.0 | | 0.2–16.0 |
| | Range of ± 2 s.d. | | 0–41.8 | | 0–14.0 |

mice show increased aneuploidy and stable chromosome aberrations compared with cells from normal mice (Bouffler et al, 1995; Wang et al, 1996).

Based on the timing of increase in growth rate coinciding with loss of both the wild-type allele and the *N*-(phosphoacetyl)-L-aspartate (PALA)-induced $G_1$ checkpoint, Tainsky et al (1995) suggested that genomic instability is a property of early-passage m/+ cells. In support of this hypothesis, introduction of mutant *TP53* under vector control into normal diploid fibroblasts induced genomic instability. However, this was observed under conditions of high levels of expression of p53 observable by Western blot analysis (Liu et al, 1996), conditions that do not apply to LFS fibroblasts. Our data argue against this hypothesis and support the suggestion derived from cells of p53-deficient mice (Livingstone et al, 1992; Harvey et al, 1993) that heterozygous cells have essentially normal chromosome stability, but an increased probability of converting to the m/– genotype that does show genomic instability.

Fibroblasts from LFS families without mutations also appear to develop more aneuploidy than normal control cells, although to a considerably lesser extent than the heterozygote strains and sometimes unaccompanied by increased structural aberrations. Although the strains studied in group E were all from cancer-affected donors, the chromosome changes are probably not a result of radio- or chemotherapy, because these were received 7 and 15 years before biopsy in cases 154MA and 159MA, respectively, and only surgery was given in case 126MA, which showed the highest levels of aneuploidy and structural chromosomes within the group. If the clinical features of LFS in these families result from altered p53 expression, despite lack of any detected mutations in *TP53*, then raised levels of aneuploidy may be an indicator of this.

### Longevity

Except for 93MA and H011, the lifespans of normal control fibroblasts ranged between 6 and 38 PD, compared with 17 and 74 PD for LF cells. There was thus a considerable overlap in longevity between the two groups, although the mean values were significantly different. Bischoff et al (1990) reported an increased lifespan in strains carrying mutations, and our results confirm that it is this group (B+C, Table 2) that has the longer lifespan. However, there is a suggestion of heterogeneity within this group giving a bimodal distribution, with four strains having 17–27 PD falling within the normal range and eight strains showing longer lifespan (44–74 PD). Loss of the wild-type *TP53* allele (m/+ to m/–) alone did not result in the immortalization of LF cells.

As expected from earlier reports (Hayflick, 1965; Goldstein et al, 1978; Allsopp et al, 1992), the longevity of normal fibroblasts showed a relatively weak negative correlation with donor age, and a similar result was obtained with LF cells (Table 3), but not for the mutation-carrying strains, possibly because of the smaller sample size. The mean population doubling time of early-passage normal fibroblasts was inversely correlated with cellular lifespan, so that cells with a short lifespan had relatively long doubling times and vice versa. A similar, but non-significant correlation held for mutation-carrying LF strains.

### Radiation resistance

The third consequence of *TP53* mutations is increased cellular resistance to ionizing radiation, particularly when administered at low dose rate (Sproston et al, 1996). A baseline for determining relative resistance of LF strains was established by determining the

© Cancer Research Campaign 1998

| Fibroblast strain | 136MA | 141MA | 135MA | 21MA/138MA | | 22MA |
|---|---|---|---|---|---|---|
| TP53 status | +/+ | +/+ | 175/+ | 175/+ | | +/+ |
| Lifespan (PD) | 24 | 17 | 74 | 44 | 48 | 22 |
| Spontaneous chromosome aberrations | n | n | ↑ | n | ↑ | |
| LDR survival | n | n | r | r | r | |
| Permanent $G_1$ arrest (%) | 62 | 77 | 41 | | 39 | |

**Figure 4** End point correlations in family 83

survival of 12 normal control strains. The wide range of sensitivity observed was similar to that observed by others (Geara et al, 1992). Apart from the most resistant strain, 85MA, all other strains gave survival values of < 30% at 3 Gy and < 10% at 6 Gy. Taking these values as the arbitrary limits of normal sensitivity, the majority of mutation-carrying strains were found to be resistant (Figure 3). An exception was 164MA (codon 245) whose survival at both doses fell within the normal range. A more stringent criterion for resistance was to set the normal limits of sensitivity at ± two standard deviations of the mean values of the control group at each dose (for example, see Table 8). When individual strains were assessed against this standard, all LF strains not carrying mutations had normal sensitivities (126MA was a borderline case), and eight mutation-carrying strains from seven donors (21MA and 138MA were from the same donor) were resistant, but heterozygous strains 131MA (codon 248), 109MA (codon 180) and 164MA (codon 245) all had normal sensitivities. These results illustrate that, although mutation-carrying strains as a group are resistant to LDR radiation, the difference between normal and LF strains is not large, and some LF strains are not significantly more resistant than normal control strains. When it occurs, radiation resistance in mutation-carrying strains would appear to be a property of the heterozygous state, as experiments were carried out on early-passage cells, before loss of the wild-type allele was observed (Tables 5 and 6; Sproston et al, 1996). Furthermore, as discussed below, radioresistance is not a universal feature of m/– LFS fibroblasts.

The present series of experiments differed slightly from our previous study (Sproston et al, 1996) in that feeder cells were not used. Nevertheless, the results confirmed the relative sensitivities of the strains used in common in the two studies. The earlier study found FH1 (codon 248) to be the most resistant of four LF strains with mutations in codons 175, 180, 220 and 248, which lie in the domain of p53 that affects specific DNA binding and protein conformation. The present data show similar high resistance in 163MA cells, from a sib of the donor of FH1, and in the duplicated strains 21MA and 138MA (codon 175). None of these strains are as resistant as 2800T (codon 234), previously reported as being resistant to HDR irradiation (Bech-Hansen et al, 1981). We also determined the radiation response of 163/29, 172MA and 161MA-F, mutation-carrying strains that have lost the wild-type allele. Clone 163/29 was resistant, while 172MA and 161MA-F had normal sensitivities. The mutations in 172MA (codon 337) and 161MA-F (codon 344) are in the carboxyterminal region that contains domains affecting tetramerization, non-specific DNA binding and possibly DNA damage recognition (Bristow et al, 1996). However, the karyotypes of all three strains are highly abnormal (Varley et al, 1996; A Spreadborough, personal communication), which makes uncertain any correlation between mutation and failure to confer resistance.

With strains derived from families with germline mutations, we have now shown that, when resistance occurs, it is in the mutation-carrying strains and not in the wild-type strains from these families, thus supporting the idea that resistance is a consequence of

© Cancer Research Campaign 1998

the *TP53* mutation. In families with no germline mutations in their *TP53* coding sequence, the presumption is that there is an undetected mutation outside the regions of the gene studied (Varley et al, 1997*b*), or in a gene regulating p53 activity, or in a gene whose product can substitute for p53 in some, but perhaps not all, ways. As the occurrence of specific types of cancer at young age (see Introduction) is a characteristic feature of the syndrome, cells from these families were classified according to whether or not their donors had cancer, on the assumption that affected individuals were most likely to be mutation carriers, although the possibility was recognized that cancer in a specific individual may be a sporadic event unrelated to p53. Radiation responses of cells from three such families were determined. Strains from family 88 fulfilled the prediction that cells from cancer-affected LF individuals might show radiation resistance through a p53-like involvement, as strain 126MA from a cancer-affected person was marginally resistant, whereas 130MA from an unaffected blood relative showed normal sensitivity. In family 81, marginal resistance (resistant at 3 Gy, normal at 6 Gy) was seen in one of two strains from cancer-affected individuals, but also in cells from an unaffected individual who was biopsied at age 45 years. The marginal resistance of 81MA is consistent with the extent of permanent $G_1$ arrest in 81MA seen in a recent study that demonstrated that the radiation resistance of LF strains is inversely correlated with the fraction of cells that are able to progress through the cell cycle when irradiated in $G_1$ (Williams et al, 1996). In the third family, family 80, two strains from affected individuals both showed normal sensitivity. Family 80 is a large family, the most dramatic cases of which show linkage to a *BRCA2* haplotype (J Heighway and G White, personal communication). Thus, cellular radiation resistance is not a consistent characteristic of LFS families without *TP53* mutations and, even when it occurs, it is difficult to demonstrate.

### Mechanistic interpretation of the data

Because of the multiple effects of p53 on cell growth and survival (recently reviewed by Ko and Prives, 1996), it is not possible at present to be precise about the mechanisms causing the effects we have observed. However, the following scenario, although simplistic, could account for most of the observed phenotypes. DNA strand breaks occur spontaneously as a result of cellular metabolism and through damaging agents such as ionizing radiation. p53 detects and is induced by the presence of double-strand DNA breaks (Nelson and Kastan, 1994) and at high concentrations causes transactivation of the cyclin-dependent kinase inhibitor p21, which is strongly implicated in permanent $G_1$ arrest (Di Leonardo et al, 1994; Williams et al, 1997) and senescence (Noda et al, 1994). We assume that p53 monitors the genome for DNA breaks (Lane, 1992) and is involved in their repair through interactions with repair proteins. As cells age, the fidelity of replication and repair may breakdown and greater numbers of DNA breaks may accumulate, resulting in an increased probability of chromosome aberrations and the transactivation of p21, leading to senescence. Abrogation of these effects by mutation could lead to increased longevity, decreased permanent $G_1$ arrest and may allow enhanced survival of genetically damaged cells after genotoxic insult (Williams et al, 1997).

The consequences of *TP53* mutation are best illustrated by family 83, from whom the greatest number of cell strains was available (Figure 4). In this family, a de novo *TP53* mutation in codon 175 occurred in generation II and was transmitted to the son in generation III. The presence of the mutation resulted in increased fibroblast lifespan, increased frequency of spontaneous structural chromosome aberrations and increased resistance associated with decreased permanent $G_1$ arrest in response to exposure to ionizing radiation.

### ACKNOWLEDGEMENTS

We thank the following colleagues for kindly providing the indicated cell strains: Professor B Gustafson (163MA, 164MA), Dr D Barnes (172MA), Professor AW Craft (FH1), Professor MC Paterson (2800T). This study was funded by the UK Cancer Research Campaign.

### REFERENCES

Allsopp RC, Vaziri H, Patterson C, Goldstein S, Younglai EV, Futcher AB, Greider CW and Harley CB (1992) Telomere length predicts replicative capacity of human fibroblasts. *Proc Natl Acad Sci USA* **89**: 10114–10118

Barnes DM, Hanby AM, Gillett CE, Mohammed S, Hodgson S, Bobrow LG, Leigh IM, Purkis T, MacGeoch C, Spurr NK, Bartek J, Vojtesek B, Picksley SM and Lane DP (1992) Abnormal expression of wild type p53 protein in normal cells of a cancer family patient. *Lancet* **340**: 259–263

Bech-Hansen NT, Blattner WA, Sell BM, McKeen EA, Lampkin BC, Fraumeni JF Jr and Paterson MC (1981) Transmission of *in vitro* radioresistance in a cancer-prone family. *Lancet* **1**: 1335–1337

Birch JM, Hartley AL, Tricker KJ, Prosser J, Condie A, Kelsey AM, Harris M, Morris Jones PH, Binchy A, Crowther D, Craft AW, Eden OB, Evans GR, Thompson E, Mann JR, Martin J, Mitchell ELD and Santibanez-Koref MF (1994) Prevalence and diversity of constitutional mutations in the *p53* gene among 21 Li–Fraumeni families. *Cancer Res* **54**: 1298–1304

Bischoff FZ, Yim SO, Pathak S, Grant G, Siciliano MJ, Giovaella BC, Strong LC and Tainsky MA (1990) Spontaneous abnormalities in normal fibroblasts from patients with Li–Fraumeni cancer syndrome: aneuploidy and immortalisation. *Cancer Res* **50**: 7979–7984

Bouffler SD, Kemp CJ, Balmain A and Cox R (1995) Spontaneous and ionising radiation-induced chromosomal abnormalities in the bone marrow of p53-deficient mice. *Cancer Res* **55**: 3883–3889

Bristow RG, Benchimol S and Hill RP (1996) Review article: The p53 gene as a modifier of intrinsic radiosensitivity: implications for radiotherapy. *Radiother Oncol* **40**: 197–223

Chen JR (1977) *In situ* detection of mycoplasma contamination in cell cultures by fluorescent Hoechst 33258 stain. *Exp Cell Res* **104**: 255–262

Di Leonardo A, Linke SP, Clarkin K and Wahl (1994) DNA damage triggers a prolonged p53-dependent $G_1$ arrest and long-term induction of Cip1 in normal human fibroblasts. *Genes Dev* **8**: 2540–2551

Dulic V, Kaufmann WK, Wilson SJ, Tlsty TD, Lees E, Harper JW, Elledge SJ and Reed SI (1994) p53-Dependent inhibition of cyclin-dependent kinase activities in human fibroblasts during radiation-induced $G_1$ arrest. *Cell* **76**: 1013–1023

Ford JM and Hanawalt PC (1995) Li–Fraumeni syndrome fibroblasts homozygous for p53 mutations are deficient in global DNA repair but exhibit normal transcription-coupled repair and enhanced UV resistance. *Proc Natl Acad Sci USA* **92**: 8876–8880

Geara FB, Peters L, Ang KK, Wike JL, Sivon SS, Guttenberger R, Callender DL, Malaise EP and Brock WA (1992) Intrinsic radiosensitivity of normal human fibroblasts and lymphocytes after high- and low-dose-rate irradiation. *Cancer Res* **52**: 6348–6352

Goldstein S, Moerman EJ, Soeldner JS, Gleason RE and Barnett DM (1978) Chronologic and physiologic age affect replicative life-span of fibroblasts from diabetic, prediabetic and normal donors. *Science* **199**: 781–782

Harvey M, Sands AT, Weiss RS, Hegi ME, Wiseman RW, Pantazis P, Giovanella BC, Tainsky MA, Bradley A and Donehower LA (1993) In vitro growth characteristics of embryo fibroblasts isolated from p53-deficient mice. *Oncogene* **8**: 2457–2467

Hayflick L (1965) The limited *in vitro* lifetime of human diploid fibroblasts. *Exp Cell Res* **37**: 614–636

Ko LJ and Prives C (1996) p53: puzzle and paradigm. *Genes Dev* **10**: 1054–1072

Lane DP (1992) Cancer. p53, guardian of the genome. *Nature* **358**: 15–16

© Cancer Research Campaign 1998

Li FP and Fraumeni JF Jr (1969) Soft tissue sarcomas, breast cancer, and other neoplasms: a familial syndrome? *Ann Intern Med* **71**: 747–752

Little JB, Nove J, Dahlberg WK, Troilo P, Nichols WW and Strong LC (1987) Normal cytotoxic response of skin fibroblasts from patients with Li–Fraumeni familial cancer syndrome to DNA damaging agents *in vitro*. *Cancer Res* **47**: 4229–4234

Liu PK, Kraus E, Wu TA, Strong LC and Tainsky MA (1996) Analysis of genomic instability in Li–Fraumeni fibroblasts with germline p53 mutations. *Oncogene* **12**: 2267–2278

Livingstone LR, White A, Sprouse J, Livanos E, Jacks T and Tlsty TD (1992) Altered cell cycle arrest and gene amplification potential accompany loss of wild-type p53. *Cell* **70**: 923–935

MacGeoch C, Turner G, Bobrow LG, Barnes DM, Bishop DT and Spurr NK (1995) Heterogeneity in Li–Fraumeni families: p53 mutation analysis and immunohistochemical staining. *J Mol Genet* **32**: 186–190

Malkin D, Li FP, Strong LC, Fraumeni JF Jr, Nelson CE, Kim DH, Kassel J, Gryka MA, Bischoff FZ, Tainsky MA and Friend SH (1990) Germ line p53 mutations in a familial syndrome of breast cancer, sarcomas and other neoplasms. *Science* **250**: 1233–1238

Mirzayans P, Aubin RA, Bonisch W, Blattner WA and Paterson MC (1995) Abnormal pattern of post-γ-ray DNA replication in radioresistant fibroblast strains from affected members of a cancer-prone family with Li–Fraumeni syndrome. *Br J Cancer* **71**: 121–1230

Nelson WG and Kastan MB (1994) DNA strand breaks: the DNA template alterations that trigger p53-dependent DNA damage response pathways. *Mol Cell Biol* **14**: 1815–1823

Noda A, Ning Y, Venable SF, Pereira-Smith OM and Smith JR (1994) Cloning of senescent cell-derived inhibitors of DNA synthesis using an expression screen. *Exp Cell Res* **211**: 90–98

Rong S, Donehower LA, Hansen MF, Strong L, Tainsky M, Jeffers M, Resau JH, Hudson E, Tsarfaty I and Van De Woude GF (1995) Met proto-oncogene is overexpressed in tumours of p53-deficient mice and tumours of Li–Fraumeni patients. *Cancer Res* **55**: 1963–1970

Sproston ARM, Boyle JM, Heighway J, Birch JM and Scott D (1996) Fibroblasts from Li–Fraumeni patients are resistant to low dose-rate irradiation. *Int J Radiat Biol* **70**: 145–150

Srivastava S, Zou Z, Pirollo K, Blattner W and Chang EH (1990) Germ-line transmission of a mutated p53 gene in a cancer-prone family with Li–Fraumeni syndrome. *Nature* **348**: 747–749

Tainsky MA, Bischoff FZ and Strong LC (1995) Genomic instability due to germline p53 mutations drives preneoplastic progression towards cancer in human cells. *Cancer Metastasis Rev* **14**: 43–48

Tsutsui T, Fujino T, Kodama S, Tainsky MA, Boyd J and Barrett JC (1995) Aflatoxin $B_1$-induced immortalisation of cultured skin fibroblasts from a patient with Li–Fraumeni syndrome. *Carcinogenesis* **16**: 25–34

Varley JM, McGown G, Thorncroft M, Cochrane S, Morrison P, Woll P, Kelsey AM, Mitchell ELD, Boyle J, Birch JM and Evans DGR (1996) A previously undescribed mutation within the tetramerisation domain of *TP53* in a family with Li–Fraumeni syndrome. *Oncogene* **12**: 2437–2442

Varley JM, Evans DGR and Birch JM (1997*a*) Li–Fraumeni syndrome – a molecular and clinical review. *Br J Cancer* **76**: 1–14

Varley JM, McGown G, Thorncroft M, Santibanez-Koref MF, Kelsey AM, Tricker KJ, Evans DGR and Birch JM (1997*b*) Germline mutations of *TP53* in Li–Fraumeni families: an extended study of 39 families. *Cancer Res* **57**: 3245–3252

Wang L, Cui Y, Lord BI, Roberts SA, Potten CS, Hendry JH and Scott D (1996) Gamma-ray-induced cell killing and chromosome abnormalities in the bone marrow of p53-deficient mice. *Radiat Res* **146**: 259–266

Williams KJ, Boyle JM, Birch JM, Norton JD and Scott D (1996) Cell cycle arrest defect in Li–Fraumeni syndrome: a mechanism of cancer predisposition? *Oncogene* **14**: 277–282

Yin Y, Tainsky MA, Bischoff FZ, Strong LC and Wahl GM (1992) Wild-type p53 restores cell cycle control and inhibits gene amplification in cells with mutant p53 alleles. *Cell* **70**: 937–948

© Cancer Research Campaign 1998